# Reducing Agricultural Waste and Losses with Nanotechnology: Shifting Paradigms in Food Safety, Produce Shelf Life, and Plant Protection

**DOI:** 10.1021/acs.jafc.4c05226

**Published:** 2024-07-10

**Authors:** Luis Cisneros-Zevallos, Mustafa Akbulut

**Affiliations:** † Department of Horticultural Sciences, Texas A&M University, College Station, Texas 77843-2133, United States; ‡ Department of Chemical Engineering, Texas A&M University, College Station, Texas 77843-3122, United States

Agriculture encompasses agronomic and high-value horticultural crops. Since the green revolution in the 1950–1970s, emphasis has been placed on increasing yields and productivity to feed a growing world population, redirecting research and financial resources to enhance total agricultural production (TAP), while less emphasis has been placed on agricultural waste and losses. In this context,
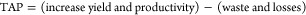
1

Over time, TAP has increased while waste and losses continue to be a major challenge. Agricultural activity has coexisted with waste and losses, and any change in reducing it will have an immediate impact on TAP and will shift this paradigm according to eq 1. Nanotechnology offers this possibility; however, it is in its infancy in its application to agriculture. Many studies have been reported, but priorities must be identified. Herein, we propose that nanotechnology efforts will have an immediate impact on TAP when priorities are directed to the main sources of waste and losses such as issues of food safety, produce shelf life, and plant protection, while there may be long-term impacts of nanotechnology on TAP when efforts are directed to increase yield and productivity (Figure 1).

**Figure 1:**
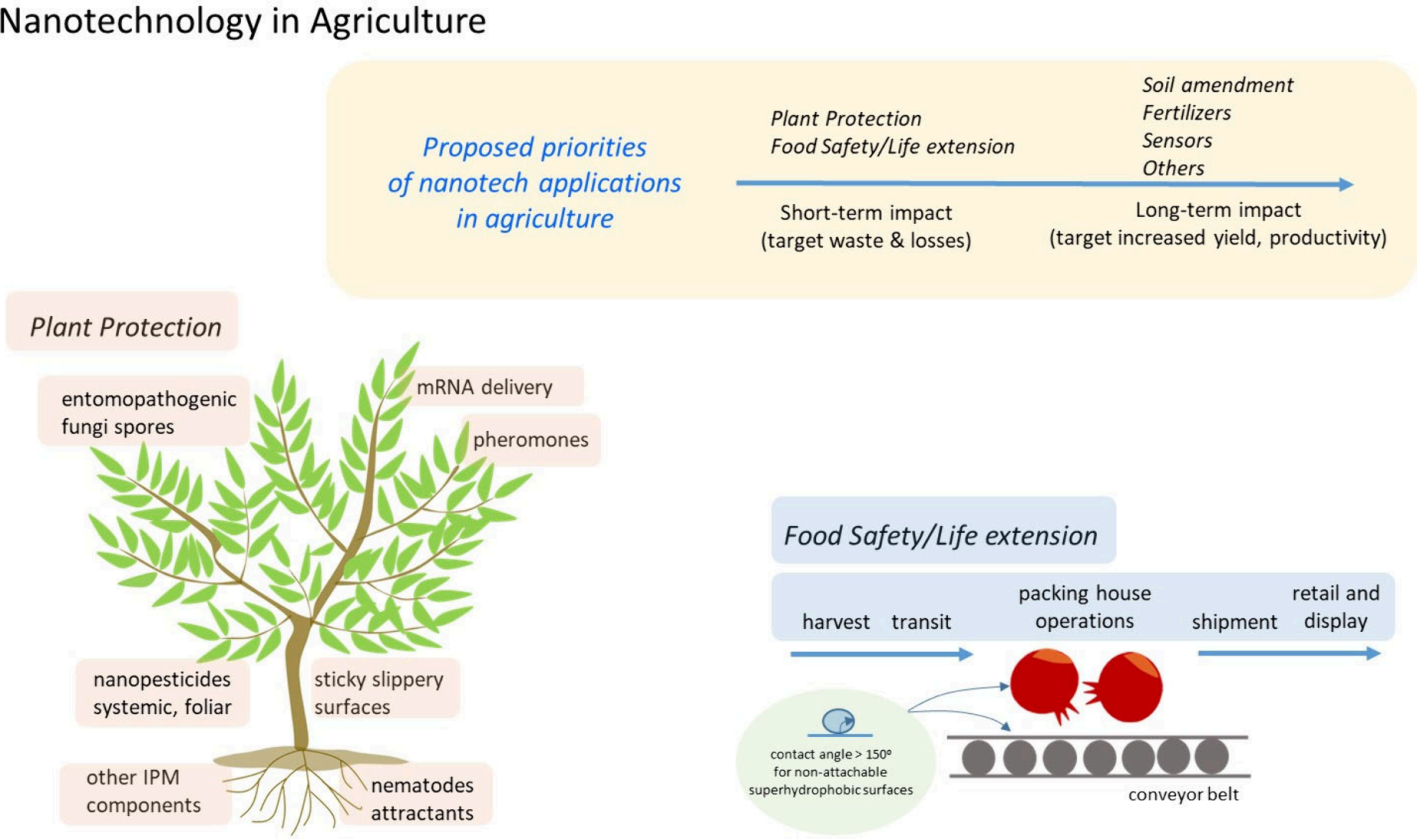
Nanotechnology in agriculture. Short-term goals focus on reducing waste and losses in plant protection (IPM components) and food preservation (superhydrophobic surfaces). Long-term objectives are to increase yield and productivity via water use efficiency, fertilizers, and sensors. Nanobased solutions targeting waste and losses will immediately impact total agricultural production (TAP) and shift the paradigm of its coexistence with current TAP.

According to the Web of Science database, 109 publications included “nanotechnology” and “agriculture” in their title or keywords from 2001 to 2012 whereas by 2024 the number had increased to 1772, indicating the scientific impact of the topic. In this Viewpoint, the concept of the coexistence of waste and loss in agriculture is revisited, emphasizing the use of nanotechnology on issues of food safety, produce shelf life, and plant protection to shift this paradigm and create an immediate impact.

## Revisiting the Concept of Plant Protection

Since the 1950s, integrated pest management (IPM) has been the preferred approach for protecting plants and reducing losses. It is implemented through IPM components that encompass a combination of tools, including biological tools, physical tools, chemicals, etc., to reduce losses to an economically acceptable level. A reduction in losses with IPM from ∼45% to 4% in potatoes and from ∼50% to <5% in sweet potatoes and cost reductions in pesticides from $1200 to $300 per hectare in asparagus have been reported.^1^ The development of chemical controls such as synthetic pesticides challenges IPM in short-term applications but affords pest resistance, creating further problems with losses in long-term scenarios. Thus, to overcome this continuing challenge posed by pesticides, the future and sustainability of IPM for plant protection and loss reduction will depend on the novel implementation of IPM components. Herein, we propose that IPM components based on nanotechnology may shift this paradigm and could include the development of nanopesticides of systemic and foliar applications, mRNA delivery systems, pheromone nanodispensers, sticky and slippery surfaces, nematode nanoattractants, adherence of entomopathogenic fungus spores, and other possible nanobased IPM components (Figure 1). Recent reports of systemic nanocarriers have shown the potential to create nanopesticides with differential internal kinetics based on nanoparticle size.^2^ Similarly, nanopesticides have shown effective killing effects in armyworm by aiding pesticide internalization and decreasing IC_50_ values compared to that of the reference pesticide.^3^ Many of the proposed nanobased IPM components have not been developed; thus, several elements would have to be studied, including the role of polarity and use of appropriate GRAS status materials, the role of nano-roughness, the polarity of targeted insect surfaces and plants surfaces (e.g., leaf, stem, root, etc.), etc.

## Revisiting the Concept of Produce Food Safety and Life Extension

Losses of fresh produce in the field and after harvest are estimated to be in the range of 5–45%. The cause of losses includes microbial to physiological disorders and is present along the chain from the field to the consumer, including harvesting operations, transit, packing house operations, shipment, and retail and display activities. Human pathogens and growth of fungi are the main sources of losses creating issues of food safety and a decrease in produce shelf life, respectively. Tools available to control these challenges and reduce risks are part of guidelines like good agricultural practices (GAP); however, losses are still present, confirming the limitations of GAPs. Herein, we propose that implementing nanobased tools may dramatically reduce losses due to food safety and shelf life issues, shifting the paradigm in this area. For instance, the development of superhydrophobic surfaces (water contact angles of >150°) for equipment in contact with produce and produce itself will eliminate the risk of microbial attachment and thus any possibility of microbe colonization and growth (Figure 1). These superhydrophobic surfaces are designed by appropriate nano-roughness and polarity conditions that can entrap nano-air pockets at the water–surface interface. Furthermore, these non-attachable surfaces or coatings can be modified to have dual or multiple functions by delivering antimicrobials.^4^ However, despite recent studies of the development of non-attachable superhydrophobic coatings for equipment^4^ and produce,^5^ several elements remain to be studied, including the role of the polarity of microbe surfaces, the polarity and roughness of equipment and fruit surfaces, the integrity and resistance to friction of the generated surfaces, the use of GRAS status materials, etc.

## Future Direction

On the basis of eq 1, the use of nanotechnology may impact TAP in short-term and long-term scenarios. For the latter, efforts to increase yield and productivity are necessary because the world global population is estimated to reach ∼9.7 billion by 2050; thus, nanobased solutions for water use efficiency, precise delivery of fertilizers, and the use of sensors of many kinds to aid in yield and productivity are a must. However, for the former as presented in this Viewpoint, nanobased efforts to reduce waste and loss will shift the paradigm of its coexistence with TAP as known at present and will create an immediate positive impact, providing a more food that is safe and has an extended shelf life. For this to happen, future studies should focus on interfacial phenomena, studying molecular interactions between surfaces and targeted microbes or insects, the role of designed nano-roughness, the identification and use of compatible materials with the food supply, and the integrity of the nanostructures generated and their safety. Furthermore, to enable these tailored nanobased solutions, all of the participants in the produce chain, farmers, processors, retailors, consumers, and regulatory agencies, must develop a win–win commitment to ensure the expected impact.
